# AZTEC—azithromycin therapy for prevention of chronic lung disease of prematurity: a statistical analysis plan for clinical outcomes

**DOI:** 10.1186/s13063-022-06604-2

**Published:** 2022-08-23

**Authors:** Tin Man Mandy Lau, John Lowe, Timothy Pickles, Kerenza Hood, Sailesh Kotecha, David Gillespie

**Affiliations:** 1 Centre for Trials Research, Cardiff University, Cardiff, UK; 2 Department of Child Health, School of Medicine, Cardiff University, Cardiff, UK

**Keywords:** Chronic lung disease of prematurity, Bronchopulmonary dysplasia, Preterm infants, Randomised controlled trial, Azithromycin, Macrolide, Statistical analysis plan

## Abstract

**Background:**

The AZTEC trial is a multi-centre, randomised, placebo-controlled trial of azithromycin to improve survival without development of chronic lung disease of prematurity (CLD) in preterm infants. The statistical analysis plan for the clinical outcomes of the AZTEC trial is described.

**Methods and design:**

A double-blind, randomised, placebo-controlled trial of a 10-day course of intravenous azithromycin (20 mg/kg for 3 days; 10 mg/kg for 7 days) administered to preterm infants born at < 30 weeks’ gestational age across UK tertiary neonatal units. Following parental consent, infants are randomly allocated to azithromycin or placebo, with allocated treatment starting within 72 h of birth. The primary outcome is survival without moderate/severe CLD at 36 weeks’ postmenstrual age (PMA). Serial respiratory fluid and stool samples are being collected up to 21 days of life. The target sample size is 796 infants, which is based on detecting a 12% absolute difference in survival without moderate/severe CLD at 36 weeks’ PMA (90% power, two-sided alpha of 0.05) and includes 10% loss to follow-up.

**Results:**

Baseline demographic and clinical characteristics will be summarised by treatment arm and in total. Categorical data will be summarised by numbers and percentages. Continuous data will be summarised by mean, standard deviation, if data are normal, or median, interquartile range, if data are skewed. Tests of statistical significance will not be undertaken for baseline characteristics. The primary analysis, on the intention to treat (ITT) population, will be analysed using multilevel logistic regression, within a multiple imputation framework. Adjusted odds ratios, 95% confidence intervals, and *p*-values will be presented. For all other analyses, the analysis population will be based on the complete case population, which is a modified ITT population. All analyses will be adjusted for gestational age and treatment arm and account for any clustering by centre and/or multiple births as a random effect.

**Conclusion:**

We describe the statistical analysis plan for the AZTEC trial, including the analysis principles, definitions of the key clinical outcomes, methods for primary analysis, pre-specified subgroup analysis, sensitivity analysis, and secondary analysis. The plan has been finalised prior to the completion of recruitment.

**Trial registration:**

ISRCTN registry ISRCTN11650227. Registered on 31 July 2018.

## Introduction

The AZTEC trial (Azithromycin Therapy for Chronic Lung Disease of Prematurity) is a randomised placebo-controlled trial to determine if a 10-day course of intravenous azithromycin improves rates of survival without chronic lung disease at 36 weeks’ postmenstrual age. This trial is supported by the National Institute of Health Research’s (NIHR) Health Technology Assessment (HTA) Programme. The study protocol has been previously published [1].

This paper describes the AZTEC trial statistical analysis plan (SAP) in advance of the trial completion. The SAP was written by the trial statisticians (TP, DG, ML) and trial manager (JL) and overseen by the chief investigator (CI, SK) and CTU director (KH). It describes the procedures to be followed for the clinical primary and secondary outcomes analyses for the trial. The final study report will follow the guidelines of the Consolidated Standards of Reporting Trials (CONSORT) for reporting randomised controlled trials [2].

The SAP has been written in accordance with the International Council for Harmonisation guidelines (E9 Statistical Principles for Clinical Trials and E3 Structure and content of clinical study reports) [3, 4] and with the Guidelines on Missing Data [5, 6].

## Background information

### Rationale

Chronic lung disease of prematurity (CLD), also known as bronchopulmonary dysplasia (BPD), is a common adverse outcome of preterm infants which results in significant respiratory morbidity in childhood [7–9] and beyond [10, 11].

Although neonatal care has improved markedly over time, the limits of viability now extend down to 23 weeks’ gestation. Since an increasing proportion of preterm-born infants who are born at an extremely early stage of lung development (canalicular/saccular) now survive, the rates of CLD have remained largely unchanged. It has been well established that these infants suffer from a “new” form of CLD with an aetiology based around lung immaturity and consequent inflammation causing parenchymal tissue damage and subsequent airway remodelling.

Studies utilising samples of airway secretions have demonstrated this inflammation peaks between 7 and 10 days after birth [12] and is exacerbated by both antenatal and nosocomial infections [8]. For many years, infection with the microbe *Ureaplasma* has been implicated in the pathogenesis of CLD through indirectly stimulating the recruitment of neutrophils to the lung and subsequent production of pro-inflammatory cytokines. A systematic review noted that infection with *Ureaplasma* was associated with increased odds of developing CLD [13].

In efforts to reduce rates of infection, early clinical studies focused on the use of macrolide antibiotics, particularly erythromycin and clarithromycin, to eradicate *Ureaplasma*. Unfortunately, the majority have been heterogeneous in design and largely underpowered thus rates of CLD in treated groups were unchanged. Additionally, the choice of macrolide, dosage, timing, and duration of therapy were not sufficiently optimised to address both the infective and pulmonary inflammatory processes that contribute to the development of CLD.

More recent trials have focused on the use of azithromycin due to its effectiveness in eradicating *Ureaplasma* but also for its unique anti-inflammatory properties [14]. The studies included in the meta-analysis by Nair and colleagues [15] demonstrated proof-of-concept that azithromycin may be effective in reducing rates of CLD, with few reports of any serious adverse reactions. Moreover, pharmacokinetic studies by the Viscardi group has also confirmed the efficacy of azithromycin in the treatment of *Ureaplasma* spp. infection [16].

Given that the current preventative strategies for CLD are largely supportive, there is clear unmet need for new therapies to improve respiratory outcomes for this vulnerable group of infants. A definitive, adequately powered randomised placebo-controlled trial of azithromycin, addressing both the infective and inflammatory aspects of the disease is required. The AZTEC trial fulfils this gap by determining if 10-day treatment with intravenous azithromycin improves rates of survival without CLD at 36 weeks’ postmenstrual age (PMA) when compared with placebo.

### Objectives of the trial

The primary objective for the AZTEC trial is to determine the effectiveness of azithromycin in increasing survival without physiologically defined CLD (moderate/severe) when compared to placebo.

The secondary clinical objectives are to determine:

- The effect of azithromycin on CLD severity and mortality rate (at 36 weeks’ PMA);
- The effectiveness of azithromycin in reducing duration of positive pressure respiratory support (i.e. conventional mechanical ventilation/high-frequency oscillatory ventilation, continuous positive airway pressure, high flow nasal cannula, number of days of oxygen dependency);
- The safety and tolerability of azithromycin;
- If colonisation with *Ureaplasma* spp. prior to randomisation modifies the treatment effect of azithromycin compared to placebo.

### Trial design

AZTEC is a multi-centre, double-blind, randomised, placebo-controlled trial of azithromycin for the prevention of chronic lung disease of prematurity in preterm infants. 796 infants < 30 weeks’ gestational age are being enrolled over a 30 month recruitment period from 28 level 3 neonatal units in the United Kingdom (UK). Trial treatment (azithromycin or placebo) is daily for 10 days, with follow-up until 36 weeks’ PMA. Serial respiratory fluid and stool samples will be collected until approximately 21 days of life.

The main trial included an internal pilot phase, which assessed the feasibility of trial participation; recruitment and consent rates; treatment compliance; and primary outcome completeness for 12 months (9 months of recruitment and 3 months of follow-up) in 5 tertiary neonatal units. The internal pilot report was reviewed by the funder, Trial Steering Committee (TSC), and the AZTEC Independent Data Monitoring Committee (IDMC) who all recommended the continuation of the pilot phase into the main trial after suggesting minor adjustments to improve recruitment rates.

### Setting

Infants are being enrolled from UK tertiary neonatal units which are designated Level 3 (regional neonatal intensive care units) and followed up at their local hospital if transferred. Infants are identified by the local clinical care team on admission and screened against the inclusion/exclusion criteria as described in the main protocol [1].

### Eligibility

#### Inclusion criteria

Infants are considered eligible for inclusion into the trial if they:

- Are born at a gestational age of < 30 weeks (including infants born as one of a multiple birth)
- Have received respiratory support for at least 2 continuous hours duration during the first 72 h of life (intubated, or by non-invasive mechanical ventilation including continuous positive airway pressure and high flow nasal cannula or a combination thereof)
- Have an indwelling intravenous line present for drug administration
- Have written informed consent provided by the parent(s)/guardian(s) within 72 h of birth
- Can receive the first dose of the investigational medicinal product (IMP) within 72 h at the latest (within 24 h of life for inborn and 48 h for outborn infants)
- Have reasonable expectation to complete 10 days of trial treatment whilst resident at the recruiting site
- Are inborn, or born at a site within the recruiting site’s neonatal network where follow-up is possible
- In the opinion of the PI, have a reasonable prospect of survival beyond 72 h of age

#### Exclusion criteria

Infants are excluded from participation in the trial if they:

- Have postnatal exposure to another systemic macrolide antibiotic (not maternal)
- Have presence of major surgical or congenital abnormalities (excluding patent ductus arteriosus or patent foramen ovale)
- Have contraindication of azithromycin as specified in the summary of characteristics of the product
- Are participating in other interventional trial that precludes participation in AZTEC

### Interventions

The IMP for AZTEC is azithromycin 500 mg (Zedbac™, Aspire pharma Ltd, UK) which is a lyophilized powder for solution for infusion in a 10-mL vial under vacuum, equivalent to 500 mg of azithromycin for intravenous administration (524 mg of azithromycin dihydrate is equivalent to 500 mg azithromycin base, citric acid, sodium hydroxide). The placebo is an empty sterile 10 mL vial under vacuum. Azithromycin powder for solution for infusion and placebo is packaged in identical 10 ml vials with the same cap, stopper, and vial. Each vial of active IMP and placebo is blinded with a tamper-evident custom-made cardboard box to ensure contents are not visible during the reconstitution process. Each participant’s Treatment Pack contains 12 vials of azithromycin or placebo. Labelling complies with Annex 13 of good manufacturing practice [17].

The dosing schedule is 20 mg/kg (10 mL/kg) azithromycin for 3 days, followed by 10 mg/kg (5 mL/kg) for 7 days, or placebo (10 days total). All doses are given via intravenous infusion (central or peripheral line) over a period of at least 1 h. Azithromycin is most likely to be effective in eradicating *Ureaplasma* spp. if administered as early as possible after birth; a 20-mg/kg dose for 3 days has recently been shown to be highly effective [16]. *Ureaplasma* spp. in the UK are generally sensitive to macrolides including azithromycin [18]. Treatment for a further 7 days was chosen to target the pulmonary inflammation which peaks between 7 and 10 days after birth [12, 19]. Sites have, therefore, been asked to aim for initiation of trial treatment at the earliest opportunity (and within 72 h after birth at the latest).

### Blinding

The vial blinding method utilises a custom cardboard carton sourced by Saint Mary’s Pharmaceutical Unit (SMPU, Cardiff, UK) similarly to a previous trial design [20]. Labelling was performed by SMPU as per a randomisation list provided by the Centre for Trials Research (CTR), Cardiff University, which was generated by an independent statistician (who had no involvement with the AZTEC trial).

### Definition of primary and secondary outcomes

#### Primary outcome

The primary outcome is defined as a composite outcome of survival at 36 weeks’ PMA and the absence of CLD (moderate/severe severity) at 36 weeks’ PMA. The derivation of this composite primary outcome requires the combination of multiple sources on the Baby Outcomes 36 Weeks’ PMA case report forms (CRF). The Baby Outcomes 36 Weeks’ PMA CRF was completed when the infant reached 36 weeks’ PMA or discharged home if earlier. Death is defined in the tick box in ‘Details about baby’ on the Baby Outcomes 36 Weeks’ PMA CRF. The severity of CLD is based on consensus criteria [21] (Table 1).

**Table 1:** Severity-based criteria of diagnosis of CLD at 36 weeks’ PMA

Received respiratory support and/or supplementary oxygen for more than 28 days, cumulatively, and the following:	
Mild CLD	• Breathing room air
Moderate CLD	• Require < 30% oxygen (or low flow 0.01–1.0 L/ min), not receiving any respiratory support
Severe CLD	• Require ≥ 30% oxygen (or low flow ≥ 1.1 L/min), still receiving respiratory support (ventilation, continuous positive airway pressure [CPAP], high flow oxygen)

**Fig. 1:**
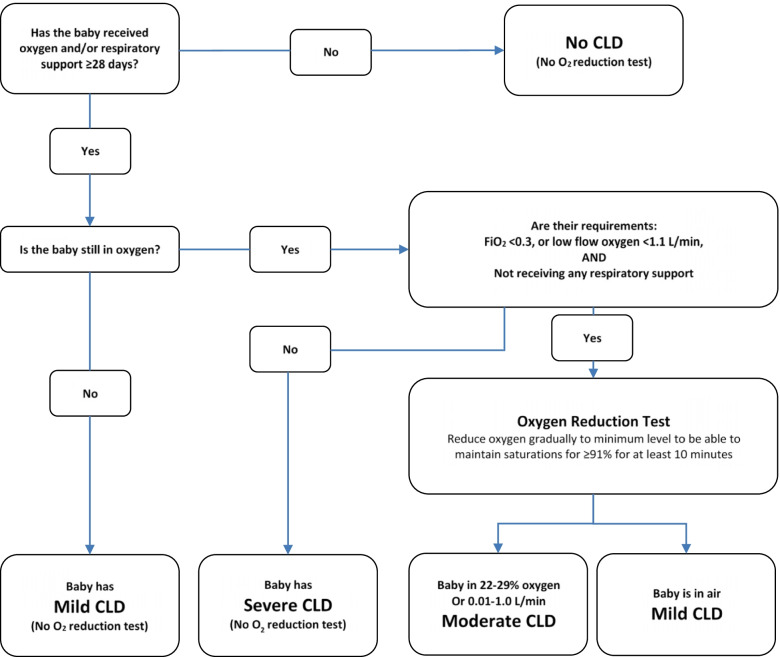
Flow diagram for assessment of CLD severity in the AZTEC trial. Modified from the original Baby-OSCAR protocol (https://www.npeu.ox.ac.uk/downloads/files/baby-oscar/protocol/Baby-OSCAR%20Protocol%20v6_171116.pdf)

Infants meeting the initial diagnosis of moderate CLD undergo a physiological test to confirm their oxygen requirement (Fig. 1). This physiological definition, initially developed by Quine and colleagues [22], has been widely used in clinical trials of neonatal lung disease [23].

#### Secondary outcomes

Incidence of the following outcomes at 36 weeks’ PMA or discharge home or death (whichever occurs earlier):

- Survival at 36 weeks’ PMA*
- Physiologically defined CLD (at moderate/severe severity)*
- Nosocomial infection confirmed microbiologically or antibiotic treatment for 5 days or more
- Severe intraventricular haemorrhage (grade III/IV)
- Necrotising enterocolitis (Bell stage II and above)
- Treatment for retinopathy of prematurity
- Treatment for patent ductus arteriosus
- Liver (max bilirubin/max AST/max ALT) and renal function (maximum creatinine’ level)
- Serious adverse events/reactions

*Survival at PMA and physiologically defined CLD are the individual components of the composite primary outcome.

Number of days of the following outcomes up to 36 weeks’ PMA or discharge home or death (whichever occurs earlier):

- Invasive ventilation by endotracheal tube required
- Non-invasive respiratory support required
- Oxygen dependency

The baseline pulmonary *Ureaplasma* colonisation will be reported in the main paper, but the later serial samples will be used to study the effects of the intervention to aid understanding and development of specific targeted therapies.

#### Process outcomes

The AZTEC trial focuses on IMP ‘initiation’ and ‘implementation’ as the two adherence elements of interest. A participant is defined as having initiated treatment during the dosing window, if ‘Yes’ under the column “IMP given today?” on the Daily log within 72 h of life has been ticked. Implementation is defined as the extent to which a participant has received their IMP as intended. This will be expressed as the number of doses the participant receives within the ten-day dosing window divided by ten, giving the total proportion of expected daily doses.

### Hypothesis framework

AZTEC will determine the superiority of azithromycin compared to placebo on survival without physiologically defined CLD (moderate/severe) at 36 weeks’ PMA.

### Sample size and power

Relevant interventional studies investigating CLD as an outcome (including studies using macrolides) in preterm infants vary in terms of their event rate. For example, the Ballard data [24] showed a survival rate without CLD of 50%, including death rate of 20% and 30% rate for development of CLD. In general, national and international studies consistently show rates of survival without CLD of 50–60% (those with lower rates are due to highly selected groups of sicker participants). Thus, adopting a conservative approach using 50% survival without developing CLD was reasonable. An effect size of 12% was deemed clinically worthwhile difference that would be convincing in the clinical arena and impact routine use. An improvement of 12% (50 to 62%) in survival without CLD with a power of 0.90 and a significance level of 5% would require 796 subjects (including a dropout rate of 10%). Since the trial involves formal assessment with an oxygen challenge test in both tertiary units and in step-down units, a dropout rate of 10% has been included in the overall target of 796 infants.

### Intervention allocation

Infants are remotely randomised using an online computerised randomisation system called Sortition created by the University of Oxford’s Primary Care Health Sciences Primary Care Clinical Trials Unit (PCCTU) (https://innovation.ox.ac.uk/licence-details/sortition-clinical-trial-randomisation-software/). The system is operational 24 h a day. Randomisation is performed by delegated members of the local neonatal trial team only after the parents/guardians of the infant have signed the consent form and the local team have completed the baseline assessments. The delegated individuals are provided with individual login details for the online system. Infants are randomised to either azithromycin or placebo using minimisation to balance treatment by site and gestational age. Infants from multiple births are randomised individually.

The randomisation code list was generated by an independent statistician at the CTR who was not involved with the AZTEC trial. The randomisation lists were generated (1:1 ratio) using block randomisation. As AZTEC is a double-blind trial, the infant’s family, clinicians, nurses, and trial team (including the data manager and statistician) are all unaware of the treatment arm to which the participant has been allocated for the duration of the trial.

Each treatment pack is labelled with a unique identification number (Pack ID). Sortition allocates a Pack ID for each participant. The participant Study IDs and Pack IDs are linked in the randomisation file, which is only accessible by an independent statistician, and the pharmacovigilance team for the purposes of unblinding for regulatory reporting.

### Data collection schedule

All assessments and data collection are completed using web-based CRFs. All data are being stored in accordance with Cardiff University and the CTR policies and procedures and in line with Good Clinical Practice (GCP). If the web-based system is not accessible, paper CRFs are being used to record data. The data is then being inputted into the web-based system once it is accessible. A summary of AZTEC study procedures and follow-up can be seen in the main protocol [1].

The CRFs to be completed are as follows:

- Consent form monitoring
- Eligibility
- Trial entry
- Follow-up contact
- Adverse reactions (AR)
- Week 1 daily log
- Week 2 daily log
- Week 3 daily log
- Transfer
- Baby outcomes up to 36 weeks’ PMA
- Baby outcomes post-36 weeks’ PMA
- Withdrawal
- Non-compliance
- Serious Adverse Event (SAE)

### Interim analyses and stopping rules

No interim analyses have been specified.

### Independent oversight committees

An IDMC, independent experts external to the AZTEC trial, and the independent TSC, are monitoring the safety of the participants and reviewing the progress of the AZTEC trial at least annually, or more often as appropriate. Any advice and issues identified during the IDMC meeting are reported to the TSC and (via the TSC) to the HTA. The committee periodically reviews the trial outcomes and progress including recruitment progress, withdrawal, data collections, IMP adherence, SAE, and any non-compliance.

During the IDMC open session, the AZTEC trial statisticians present the trial progress report to the committee without treatment allocations. The committee then in a closed session (excluding any AZTEC team members) reviews the unblinded data prepared by an independent statistician with no involvement with the AZTEC trial. The IDMC chair informs the TSC Chair of their conclusions including reporting of any issues identified during both the open and closed session of the meeting. The TSC, consisting of independent members and CI, review and address any of IDMC’s concerns before responding formally on how any concerns will be addressed. The IDMC has the responsibility to recommend continuation or not of the trial based on their review of the data including recruitment rates, etc.

### Trial reporting

Analysis of the trial outcomes will be conducted following the completion of the last follow-up of the last recruited infant, data cleaning, and the final hard locking of the database.

### Non-compliances of GCP and/or protocol

Non-compliances of GCP and/or Protocol will be categorised as either a deviation, violation, or serious breach and will be listed in the final report. These are defined as:

- *Deviation*: A planned or unplanned departure from the protocol or GCP that does not increase risk or decrease benefit; or does not have a significant impact on the participant’s rights, safety, or welfare; and/or on the integrity of the data.
- *Violation*: Unplanned departure from the protocol or GCP that increases the risk or decreases the benefit; or may have an impact on the participant’s rights, safety or welfare; and/or on the integrity of data.
- *Serious breach*: A breach of the protocol or GCP which is likely to affect to a significant degree.

- The safety or physical or mental integrity of the trial participants; or
- The scientific value of the Trial.

In the event of non-compliance, the site principal investigators will report to the CTR in writing as soon as they become aware of it. The CTR will assess the nature and severity of any issues of non-compliance, in terms of the participant right, safety, welfare, and data integrity, in accordance with the CTR standard operating procedures (SOPs).

## Analysis populations

### Population definitions

#### Intention to treat population

The ITT population will include all randomised infants. For infants with missing data, imputation will be performed (see the “Missing data” section).

#### Modified intention to treat population

The modified ITT population will include all randomised infants for whom outcome data is available.

### Descriptive analyses

#### Screening data

Screening data will be presented by the recruitment site and in total.

- Number of screened infants
- Number and proportion of those screened who were considered eligible
- Number and proportion of those considered eligible who were consented
- Number and proportion of those consented who were randomised

#### Eligibility

The number and proportion failing each inclusion criterion or failing into each exclusion criterion will be tabulated by the recruitment site.

#### Recruitment

In addition to the summaries provided for screening, the below data will be summarised for each recruitment site and in total and will be summarised in a CONSORT flow diagram.

- Received the randomised allocation
- Did not receive the randomised allocation
- Were lost to follow-up
- Discontinued the intervention

#### Withdrawal

Withdrawal is defined at the following levels and will be reported by the recruitment site, treatment arm and in total:

- Withdrawal of trial treatment
- Withdrawal from samples
- Withdrawal from follow-up assessments
- Withdrawal of consent to all of the above but permitting use of already data collected and medical records can be examined
- Withdrawal of consent for the entire trial including withdrawal from use of any already collected data

#### Timing of withdrawal

The stages throughout the trial at which withdrawals will be summarised by the recruitment site, treatment arm, and in total:

- Prior to randomisation
- Prior to commencing treatment
- During treatment period (up to Day 10 of treatment)
- In the process of, or during, transfer to a step down unit
- 36 weeks’ PMA or discharge home or death (whichever occurs earlier)
- Discharge home or death (whichever occurs earlier) post 36 weeks’ PMA

#### Reason for withdrawal

The following reasons for withdrawal are collected and will be summarised by the recruitment site, treatment arm, and in total:

- Intolerance to medication
- Withdrawal of consent for treatment by the parent(s)/guardian(s)
- Any alteration in the infant’s condition which justifies the discontinuation of the treatment in the Investigator’s opinion
- Non-compliance

#### Baseline data

The following data are being collected at trial entry (pre-randomisation) and will be summarised by treatment arm and in total: infant’s sex, gestational age at birth, birthweight, mode of delivery, cause of preterm birth, multiple babies, place of birth, mother’s ethnicity, mother’s antenatal corticosteroid treatment, mother receive antibiotics antenatally within 5 days before delivery, and mother received magnesium sulphate for neuroprotection antenatally.

Categorical data will be summarised by numbers and percentages. Continuous data will be summarised by mean, standard deviation and if data are normal, or median, interquartile range and if data are skewed. Tests of statistical significance will not be undertaken for baseline characteristics; rather, the clinical importance of any imbalance will be noted as recommended by the CONSORT 2010 statement [25].

#### Primary analyses

The primary outcome is defined as a composite outcome of survival at 36 weeks’ PMA and the absence of CLD (moderate/severe severity) at 36 weeks’ PMA. Infants will be classified by the severity of CLD as defined in Fig. 1. In instances where an oxygen reduction test had not been performed on an otherwise eligible infant, a diagnosis of moderate CLD will be assigned.

The primary outcome will be analysed using a random-effects multilevel logistic regression, within a multiple imputation framework. The analysis will adjust for treatment arm and gestational age (< 28 weeks or 28 to < 30 weeks) and account for both clustering of both multiple births and participants within the recruitment sites. Results will be presented as adjusted odds ratios, 95% confidence intervals, and *p*-values. Should convergence difficulties be encountered when fitting both multiple births and centres as levels, the primary analysis will drop multiple births as a level.

#### Secondary analyses

Dichotomous secondary outcomes will be analysed using logistic regression and reported as adjusted odds ratios, 95% confidence intervals, and *p*-values.

Secondary outcomes investigating the number of days of respiratory support will be analysed using survival analysis allowing for competing risk of death. Secondary outcomes liver (max bilirubin/max AST/max ALT) and renal function (maximum creatinine’ level) will be analysed using linear regressions after transformation if required. These models will be multilevel to account for any clustering effects of centre and also multiple births within the same pregnancy and will be reported as adjusted hazard ratios, 95% confidence intervals, and *p*-values.

Statistical tests will not be used on serious adverse events/reactions: these outcomes will use descriptive statistics only.

#### Pre-specified subgroup analyses

Pre-specified subgroup analyses on the primary outcome and its components will be based on:

- Presence or absence of *Ureaplasma* spp. colonisation at baseline (as measured prior to randomisation). This may not be a true reflection of *Ureaplasma* spp. colonisation at baseline, as *Ureaplasma* spp. is difficult to detect and may not be detectable until sometime after randomisation;
- Infant being inborn (infants delivered in a tertiary neonatal unit where the AZTEC trial was conducted) or outborn (infants transferred to the tertiary neonatal unit where the AZTEC trial was conducted);
- Gestational age (< 28 weeks or 28 to < 30 weeks);
- Recruiting centre;

These analyses will be undertaken by extending the primary outcome analysis and including a sub-group by treatment arm interaction term. Estimates from the statistical models (main effects and interaction terms) will be presented alongside 95% confidence intervals and *p*-values. For the recruiting centre subgroup analysis, two models will be compared: the original primary outcome model with a random intercept by centre, and a random intercepts and random slopes model with random intercepts by centre and slopes by treatment. Models will be formally compared using the likelihood ratio test, with evidence of a differential treatment effect by centre concluded if the random slopes model leads to a statistically superior model fit. A treatment effects by centre plot will be included in the final report to visualise any variability in treatment effect.

#### Sensitivity analyses

To explore the impact of departures from randomised treatment on our primary analysis, the complier average causal effect will be estimated, with two definitions of “complier” considered:

- Participants who initiate within 72 h of life (as defined as ‘initiation’ in the “Process outcomes” section);
- Proportion of IMP taken during the dosing window (as defined as ‘implementation’ in the “Process outcomes” section). This second analysis will be used to explore the impact of an increase in dose on our primary outcome.

Instrumental variable methods will be used to conduct these analyses, which will use randomisation as an instrument.

Our primary analysis will be valid under a missing at random (MAR) assumption. However, we will conduct a series of sensitivity analyses exploring different modelling assumptions made with regards to missing outcome data:

- The primary outcome model will be re-fitted directly controlling for whether the participant was transferred from their recruiting site prior to the primary outcome assessment;
- In order to explore the robustness of our findings to assuming babies with a missing oxygen reduction test (for those in whom one was indicated) had moderate CLD, the primary analysis setting will be re-fitted by setting these infants to missing and impute their outcome.
- To explore the impact of deviations from the MAR assumption, further analysis will be conducted on complete case population (valid under a missing completely at random (MCAR) assumption), as well as a series of sensitivity analyses, within the multiple imputation framework, exploring the robustness of conclusions based on an MAR assumption. This will be achieved by using delta-based imputation whereby an offset term will be added to the expected value of the missing data to determine the deviation which would need to be observed within participants who did not provide data in order to alter trial conclusions [5].
- For complete cases, the population will be altered to include just those with a valid response (Yes or No) to the primary outcome.

#### Significance levels and *p*-values

The significance level is set at *α* = 0.05 (two-sided). All estimates will be followed by a two-sided 95% confidence interval. No adjustment for multiplicity will be made.

#### Missing data

The imputation model for primary analysis will use the treatment arm and gestational age variables, as well as whether or not the participant was transferred from their recruiting site prior to the primary outcome assessment (a likely source of missing data which may also be related to the primary outcome). Our analysis will address the clustering by the recruitment sites and multiple births within the same pregnancy by including indicator variables for multiple births and centres within the imputation model [26, 27]. The augment option will be used to avoid perfect prediction of the outcome by the variables included in the imputation model [28]. The number of imputations datasets created from which the analysis will be averaged over will be greater than or equal to the percentage of incomplete cases (defined as a case missing the primary outcome) out of all those randomised [29].

Missing data will remain missing for all secondary outcomes.

#### Statistical software employed

The latest versions of IBM SPSS Statistics (IBM, state, US) and Stata (Company name, state, US) will be used for data manipulation, descriptive statistics, and all other analyses (including but not limited to logistic regression, ordinal regression and competing risks survival analyses).

#### Safety data

Serious adverse events and reactions will be recorded and reported as secondary outcomes.

#### Additional exploratory analysis

No additional exploratory analyses are planned. However, any analyses not specified in the analysis protocol and in the SAP will be documented as post hoc analysis in the final report.

#### Deviation from analysis described in protocol

None yet.

## Data Availability

The SAP along with all other documents relating to the analysis of this trial will be stored in the Statistical Analysis Master File on a secure server at the CTR.

## Abbreviations

AR: Adverse reactions
BPD: Bronchopulmonary dysplasia
CI: Chief Investigator
CLD: Chronic lung disease of prematurity
CONSORT: Consolidated Standards of Reporting Trials
CPAP: Continuous positive airway pressure
CRF: Case report form
CTR: Centre for Trials Research
GCP: Good Clinical Practice
HTA: Health Technology Assessment
IDMC: Independent Data Monitoring Committee
IMP: Investigational medicinal product
ITT: Intention to treat
MAR: Missing at random
MCAR: Missing completely at random
NIHR: National Institute of Health Research
PCCTU: Primary Care Clinical Trials Unit
PMA: Postmenstrual age
SAE: Serious adverse event
SAP: Statistical analysis plan
SMPU: Saint Mary’s Pharmaceutical Unit
SOP: Standard operating procedures
TSC: Trial Steering Committee
UK: United Kingdom
